# Editorial

**Published:** 1842-06-11

**Authors:** 


### THE MEDICAL EXAMINER.

### PHILADELPHIA, JUNE 4, 1842.

The Naval Medical Board, composed of Surgeons W. P. C. Barton, (President,) John A. Kearney, Waters Smith, Thomas Dillard, and W. S. W. Ruschenberger, (Members,) adjourned on Monday, the 6th of June, after a session of two months.

The following is a list of the successful candidates, with their places of residence, the names of the schools of which they are graduates in medicine, and the subjects of their theses before the Board.

Richard W. Leecock, Assistant Surgeon, U. S. Navy, was examined and approved for promotion. Subject of his thesis before the Board, Traumatic Hæmorrhage.

### CANDIDATES PASSED FOR ADMISSION INTO THE NAVY AS ASSISTANT SURGEONS.

| *Name.* | *Residence.* | *Where Graduated.* | *Subject of Thesis before the Board.* |
|---|---|---|---|
| Wm. S. Bishop. | Pennsylvania. | Jefferson College. | Delirium Tremens. |

| *Name.* | *Residence.* | *Where Graduated.* | *Subject of Thesis before the Board.* |
|---|---|---|---|
| Saml. M. Edgar. | Tennessee. | University of Pennsylvania. | Geology of Tenn. and Ky. |
| Jos. Wilson, Jr. | Pennsylvania. | do. | The Pulse. |
| Charles Eversfield. | Maryland. | University of Virginia. | Diseases of the Chest. |
| Elisha K. Kane. | Pennsylvania. | University of Pennsylvania. | Uses of the Pancreas. |
| Edward Hudson. | Pennsylvania. | do. | Asphyxia. |
| Richard McSherry. | Virginia. | do. | Bites of rabid animals and venomous reptiles. |
| Wm. Pitt Canning. | Massachusetts. | Not graduated. | Burns and their Treatment. |
| Ephraim J. Bee. | New Jersey. | University of Pennsylvania. | Gangrene. |
| J. L. Burtt. | Ohio. | do. | Poisoning by Arsenic. |
| John T. Bartow. | Georgia. | Georgia Medical College. | Diseases of the Rectum. |
| Alfred C. Holt. | Georgia. | Jefferson College. | Causes which modify the operation of medicines. |
| James Hamilton. | Maryland. | University of Maryland. | Domestic Chemistry. |
| Charles H. Oakley. | New York. | Col. of Phys. & Surg. N. Y. | Diseases of the Eye. |
| Reuben N. Baer. | Pennsylvania. | Pennsylvania College. | Erysipelas. |

The order under which the Board acts, requires it to direct its attention "to moral character, as well as to scientific and professional attainments; and it will be its duty to make the examination *full*, *minute*, and *rigid*. The candidates must be twenty-one years of age, and not over twenty-eight; of sound health and constitution; and in *all respects* able to perform the duties of assistant surgeon in the navy."

A circular from the navy department, addressed to candidates, states:—"The Board rigidly scrutinizes the pretensions of each candidate; taking into consideration his physical qualifications and moral habits, as well as his professional acquirements; and reports favourably upon no case admitting of a reasonable doubt, as the health and lives of the officers, seamen, and marines, are objects too important to be committed to ignorant and incompetent hands. The Board reports the relative merit of the candidates, as shown by the examination. Those of whose qualifications the Board is satisfied, are appointed assistant surgeons, as their services are required."

The branches upon which candidates are examined are, Materia Medica, Toxicology and Medical Jurisprudence, Anatomy, (Special and Surgical,) Physiology, Surgery, Chemistry, the Practice of Medicine, and occasionally the Collateral Sciences, according to the circumstances of the individual candidate. And the Board looks closely to primary education, as being, in a measure, the foundation upon which medical education must rest.

The subject of the thesis is assigned by the Board; and it is immediately written by the candidate in an adjoining apartment. A single sheet of foolscap paper is given to each one, so that the opportunity of copying is cut off; nothing but previous knowledge and a well trained habit of thought, will enable the candidate to produce a reasonably respectable treatise, written under these difficulties and disadvantages. It is a pretty sure, though severe test of the candidate's knowledge, his readiness, and habit of thought.

The candidates are required to exhibit their knowledge of bandaging, and dressing of fractures, on casts or upon a living subject, in the presence of the Board.

Thirty-two candidates were examined by the Board.

| | | | |
|---|---|---|---|
| Of this number | 11 | are graduates of the | University of Pennsylvania. |
| " | 3 | " " | Jefferson Medical College. |
| " | 2 | " " | Bowdoin College. |
| " | 2 | " " | University of Maryland, Baltimore. |
| " | 2 | " " | Pennsylvania Medical College. |
| " | 2 | " " | University of N. Y. (New School.) |
| " | 1 | " " | Columbia College, D. C. |
| " | 1 | " " | University of Virginia. |
| " | 1 | " " | Medical College, Charleston, S. C. |
| " | 1 | " " | Col. of Phys. and Surgeons, N. Y. |
| " | 1 | " " | Washington University, Baltimore. |
| " | 1 | " " | Yale College. |
| " | 1 | " " | Georgia Medical College. |
| " | 1 | " " | Medical School, Harvard. |
| " | 1 | " " | Medical College of Louisiana. |
| " | 1 | " " | Not yet graduated. |
| | 32 | | |

Though passing the ordeal of the Navy Board is doubtless highly honourable to the candidate, censure or discredit is not to be indiscriminately attached to those who may be rejected. It must be borne in mind that, not only professional knowledge and a " general fitness" are required in those who present themselves, but that their health and physical qualifications are narrowly scanned.

We have heard the opinion expressed by a distinguished member of the Board, that some of our most respectable and even eminent practitioners might be rejected on this score of " general fitness"—or under the concluding portion of the Department's instructions to the Board—to select only such as are in *all respects* able to perform the duties of assistant surgeon in the navy.

---

*New England Quarterly Journal of Medicine and Surgery.*—The first number of this journal has made its appearance. It is edited by Drs. C. E. Ware and S. Parkman, assisted by an able and practised corps of contributors. The present number is most creditable in every respect, including very neat 'externals,' to the parties concerned, and we have no hesitation in predicting that the New England Journal will meet with distinguished success, and do good service to the cause of medical science. We have great pleasure in receiving so valuable an addition to our list of exchanges.

*New York Medical Gazette.* This journal will in future be edited by Professor C. R. Gilman, assisted by Dr. Geo. Wilkes and Professor Robert Watts, Jr. It has heretofore been conducted with much ability, pursuing an independent course in criticism, and in no instance deviating from gentlemanly or professional propriety. The names of the editors, now announced, are a guarantee that it will maintain its present high character.

---

*Appointments to Professorships.*—Dr. Kirtland, Professor of the Theory and Practice of Physic in the Medical College of Ohio, has resigned this chair, and been appointed to teach the same branches in the Willoughby Medical School.

Dr. Robert Bridges has been appointed Professor of Chemistry in the Philadelphia College of Pharmacy, in the place of Dr. Wm. R. Fisher, resigned.

Dr. Joseph Roby has been appointed Professor of Anatomy in the University of Maryland.

Dr. Murphy, of Dublin, has been appointed successor to the late Dr. Davis, as Professor of Midwifery in University College, London.

